# An Analytical Study to Determine the Severity of COVID-19 Among Smokers

**DOI:** 10.7759/cureus.23802

**Published:** 2022-04-04

**Authors:** Kirubhakaran Kanakaraju, Harshavarthanan Vanoli, Hamsavardhini Rajenthrakumar, Arunkumar Asokan, Rangabashyam Seetharaman Ranganathan

**Affiliations:** 1 General Medicine, Vinayaka Mission Kirupananda Variyar Medical College and Hospital, Salem, IND

**Keywords:** india, ct severity scores, mortality, smoking, covid-19

## Abstract

Introduction

In December of the year 2020, the SARS-CoV-2 virus was discovered in Wuhan, China. It extended to over 180 nations around the world. It can manifest in patients who are asymptomatic to those who are symptomatic, with symptoms ranging from anosmia to severe respiratory distress syndrome. It affects both men and women. The existence of comorbidity is also linked to a significant worsening of the infection. Despite the fact that the principal consequences of coronavirus disease 2019 (COVID-19) damage the lungs, the prevalence of current smokers among COVID-19 hospitalized patients has repeatedly been observed to be lower than the prevalence of smokers in the general community. As a result, the evidence from various studies appears to cast doubt on active smoking as a risk factor for COVID-19 pneumonia. Thus, with this background, this study has been conducted with the aim of assessing the influence of smoking as a risk factor for COVID-19 mortality.

Methodology

An observational study was conducted in a tertiary care center in Tamil Nadu for a period of three months (April 2021 to June 2021). The study participants were all the patients admitted to the COVID-19 ward of the department of general medicine during the study period. Those who were not willing to participate in the study were excluded. The questionnaire contains variables including socio-demographic characteristics, vitals, and investigations, and the outcome variable was death due to COVID-19. The data obtained were entered in Microsoft Excel (Microsoft Corporation, Redmond, WA) and the results were analyzed using SPSS version 21 (IBM Corp., Armonk, NY).

Results

About 401 individuals participated in the study. The mean age, COVID-19 Reporting and Data System (CO-RADS) score, and CT severity score of the study participants were 50 years, 4.91, and 10.61, respectively. About 63.3% of participants were males, about 92% have not been vaccinated, about 91.8% have a CO-RADS score of 5, about 45.1% were smokers, and about 15.7% have died despite effective treatment. When looking for adverse outcomes, being male (p = 0.047), non-vaccinated for COVID-19 (p = 0.042), and being a smoker (p = 0.008) were the factors that showed statistical significance.

Conclusion

The mortality due to COVID-19 is high among smokers than non-smokers with statistical significance. Thus, before admitting COVID-19 patients, to classify the patients as mild, moderate, and severe, the risk factor of the habit of smoking can be added. Cigarette smoke is harmful to the lungs in a variety of ways, and further research is needed to understand why there is such a low proportion of current smokers among COVID-19 patients in hospitals. The impact of current smoking on SARS-CoV-2 infection is a delicate and complex topic that should be thoroughly investigated before sending out potentially misunderstood signals.

## Introduction

In December of the year 2020, the SARS-CoV-2 virus was discovered in Wuhan, China. It was extended to almost all countries around the world. As of June 2021, about 18 crores of coronavirus disease 2019 (COVID-19) cases and 39 lakhs deaths were reported globally. It can manifest in patients who are asymptomatic to those who are symptomatic, with symptoms ranging from anosmia to severe respiratory distress syndrome. It has an effect on both men and women. The existence of comorbidity is also linked to a significant worsening of the infection [1]. SARS-CoV-2 predominantly infects alveolar epithelial cells in the lungs. It can cause acute respiratory distress syndrome and, on rare occasions, multiorgan failure. The presence of underlying respiratory impairments, such as chronic obstructive pulmonary disease (COPD) or smoking history, has an impact on the disease's outcome. Many case studies have found that active smokers have a high frequency of severe COVID-19 [2-4]. Smoking has also been identified as a risk factor for COVID-19's worsening progression. When compared to never smokers, current and former smokers are more likely to get community-acquired pneumonia. Many studies have found that smokers have a twice-as-high risk of COVID-19 infection. COVID-19 is no exception to the rule that smokers are more susceptible to lung infection [1,5]. Tobacco use has been linked to a variety of respiratory disorders, and significant data have shown that it has a deleterious impact on lung health. Smoking weakens the immune system and reduces its ability to respond to infections, rendering smokers more susceptible to infectious diseases [6,7]. According to earlier studies, smokers are twice as likely as non-smokers to get influenza, have more severe symptoms, and had a higher mortality rate during the previous Middle East respiratory syndrome-related coronavirus (MERS-CoV) pandemic. Despite the fact that COVID-19's principal consequences damage the lungs, the percentage of current smokers among COVID-19 hospitalized patients has repeatedly been observed to be lower than the prevalence of smokers in the general community. As a result, the evidence from various studies appears to cast doubt on active smoking as a risk factor for COVID-19 pneumonia. Thus, with this background, this study has been conducted with the aim of assessing the influence of the habit of smoking on mortality due to COVID-19.

## Materials and methods

Study design and study period

An observational analytical study was done in a tertiary care hospital in Salem, Tamil Nadu, for a period of three months (April 2021 to June 2021).

Inclusion criteria

All the patients who were admitted to the COVID-19 ward during the study period and diagnosed with COVID-19 infection either by reverse transcription-polymerase chain reaction (RT-PCR) for COVID-19 or chest CT findings were included in the study.

Exclusion criteria

The patients who were not willing to participate in the study and patients who were extremely ill during admission were excluded from the study.

Sampling technique

The eligible study participants were included by a convenient sampling technique. With the assumption of COVID-19 prevalence as 50% and absolute error as 5%, the estimated sample size was 400.

Data collection

The data were collected after getting ethical clearance from the Institutional Ethical Committee of Vinayaka Mission Kirupananda Variyar Medical College and Hospital, Salem (approval number: VMKVMC&H/IEC/21/112) by interviewing the patients using a semi-structured questionnaire and visualizing and cross-checking the hospital records.

Study tool

The questionnaire contains variables including socio-demographic characteristics such as age, religion, and gender, vitals and Investigations, CT severity scores, and RT-PCR test results, and the outcome variable was death due to COVID-19.

Statistical analysis

The data obtained were entered in Microsoft Excel (Microsoft Corporation, Redmond, WA) and the results were analyzed using SPSS version 21 (IBM Corp., Armonk, NY). The continuous variables were represented as mean with standard deviation and the frequency variables were represented as percentages. To find the test of significance between the frequency variables, the chi-square test was used. The P-value of less than 0.05 was considered statistically significant with a 95% confidence level.

## Results

A total of 401 individuals participated in our study. The mean age, COVID-19 Reporting and Data System (CO-RADS) score, and CT severity score of the study participants were 50 years, 4.91, and 10.61, respectively, as shown in Table 1.

**Table 1:** Distribution of study participants according to their age, CT CO-RADS score, and CT severity score (n = 401). CO-RADS: COVID-19 Reporting and Data System.

	Age (in years)	CT CO-RADS	CT severity score
Mean	50.74	4.91	10.61
Median	51.00	5.00	10.00
Mode	60	5	8
Standard deviation	14.545	0.675	5.045
Minimum	20	0	0
Maximum	92	6	28
Interquartile range	40.00-61.00	5.00-5.00	7.00-14.00

Among the study participants, about 63.3% were males, about 92% have not been vaccinated, about 91.8% have a CO-RADS score of 5, about 45.1% were smokers, and about 15.7% have died despite effective treatment. The baseline characteristics of the study population are shown in Table 2.

**Table 2:** Baseline characteristics of the study participants (n = 401). RT-PCR: reverse transcription-polymerase chain reaction; CO-RADS: COVID-19 Reporting and Data System.

S. No	Baseline characteristics		Frequency	Percent
1	Gender	Female	147	36.7
		Male	254	63.3
2	COVID-19 vaccination status	No	369	92.0
		Yes	32	8.0
3	COVID-19 vaccination	1st dose	20	5.0
		2nd dose	12	3.0
		No	369	92.0
4	RT-PCR	Negative	31	7.7
		Not done	252	62.8
		Positive	118	29.4
5	CT CO-RADS	0	6	1.5
		3	3	0.7
		4	13	3.2
		5	368	91.8
		6	11	2.7
6	Mortality	No	338	84.3
		Yes	63	15.7
7	Blood group	A negative	16	4.0
		A positive	14	3.5
		AB negative	3	0.7
		AB positive	33	8.2
		B negative	16	4.0
		B positive	30	7.5
		O negative	16	4.0
		O positive	273	68.1
8	Habit of smoking	No	220	54.9
		Yes	181	45.1

When comes to mortality, being male (p = 0.047), not being vaccinated for COVID-19 (p = 0.042), and having the habit of smoking (p = 0.008) have a statistically significant association with adverse clinical outcomes (death). Similarly, individuals having an O-negative blood group have a statistically significant association with mortality due to COVID-19. These associations have been shown in Tables 3, 4

**Table 3:** Association between the risk factors and mortality among the study participants due to COVID-19 (n = 401). Statistically significant values are in bold. RT-PCR: reverse transcription-polymerase chain reaction.

S. No				Mortality		Total	Chi-square value	P-value
				No	Yes			
1	Gender	Female	Count	131	16	147	4.082	0.047
			%	89.1%	10.9%	100.0%		
		Male	Count	207	47	254		
			%	81.5%	18.5%	100.0%		
2	COVID-19 vaccination status	No	Count	307	62	369	4.160	0.042
			%	83.2%	16.8%	100.0%		
		Yes	Count	31	1	32		
			%	96.9%	3.1%	100.0%		
3	RT-PCR	Negative	Count	26	5	31	0.215	0.0898
			%	83.9%	16.1%	100.0%		
		Not done	Count	211	41	252		
			%	83.7%	16.3%	100.0%		
		Positive	Count	101	17	118		
			%	85.6%	14.4%	100.0%		
4	Habit of smoking	No	Count	195	25	220	6.955	0.008
			%	88.6%	11.4%	100.0%		
		Yes	Count	143	38	181		
			%	79.0%	21.0%	100.0%		

**Table 4:** Association between the individual's blood group and mortality among the study participants due to COVID-19 (n = 401). * Fisher's exact test value.

			Mortality		Total	Chi-square value	P-value
			No	Yes			
Blood group	A negative	Count	15	1	16	85.892^*^	<0.001
		%	93.8%	6.3%	100.0%		
	A positive	Count	13	1	14		
		%	92.9%	7.1%	100.0%		
	AB negative	Count	2	1	3		
		%	66.7%	33.3%	100.0%		
	AB positive	Count	16	17	33		
		%	48.5%	51.5%	100.0%		
	B negative	Count	9	7	16		
		%	56.3%	43.8%	100.0%		
	B positive	Count	26	4	30		
		%	86.7%	13.3%	100.0%		
	O negative	Count	3	13	16		
		%	18.8%	81.3%	100.0%		
	O positive	Count	254	19	273		
		%	93.0%	7.0%	100.0%		

## Discussion

In the present study, the mean age of the study participants was 50.74 years. In contrast to our study, a study done by Bhattacharya et al. (2021) in New Delhi among 55 hospitalized patients diagnosed with COVID-19 states that the mean age was 37 years. This difference may be due to the difference in the sample size and the difference in the study settings [8]. The present study states that the majority of the study participants were males and being male has a statistically significant association with mortality due to COVID-19. Similar results were given by a study done by Dalal et al. (2021) in Italy to compare the case fatality rate (CFR) in gender among 1656 COVID-19 deaths through secondary data, stating that women have lower CFR than the male gender [9]. Another study done by Skrip et al. (2020) in the USA using secondary data analysis states that the mortality was predominantly in males, which is 61% [10]. The present study states that individuals not been vaccinated have a statistically significant association with mortality due to COVID-19. Similar results were given by another study done by Bhattacharya et al. (2021) in New Delhi among 55 hospitalized patients diagnosed with COVID-19, which states that the odds of getting ICU admission and death among fully vaccinated individuals was 0.07. Thus, there is a 70% reduction in mortality given by full vaccination [8]. Another study was done in England by Lopez Bernal et al. (2021) with the aim of comparing the available vaccines against mortality due to COVID-19, which states that all vaccines have similar effects in preventing mortality [11]. Our study states that the prevalence of smoking among the study participants is 45.1% and there is a statistically significant association between the habit of smoking and mortality. A study was done by Gaiha et al. (2020) in California with the aim of assessing the association between youth smokers and COVID-19 diagnosis, which states that this disease is six times more prevalent in smokers [12]. But in contrast to our study, a study done by Tsigaris et al. (2020) in Europe concluded that there is a negative association between smoking prevalence and COVID-19 infection [13]. Thus, this discussion needs further research. Tobacco smoke causes epigenetic changes in the bronchial epithelium, which results in mucous (goblet) cell metaplasia. Because goblet cells are a key source of angiotensin-converting enzyme 2 (ACE2) in the lungs, this could help to explain why smokers' lungs have higher amounts of ACE2. Goblet cells, on the other hand, are the primary generator of mucous, which acts as the first line of defense against inhaled pathogens, preventing pathogen entry and infection.

Strength and limitation

Though this study takes a convenient sampling, an ample number of study participants were included. The data were collected and analyzed by a single investigator. This eliminates the inter-observer bias. All data were collected from the case sheets and were counterchecked with the computerized hospital records, which increases the validity of the data. Though the habit of smoking is acquired through a questionnaire, a subjective method, these data have certain limitations (may lead to social desirability bias). This is an observational cross-sectional study, thus the association found in this study was not needed for causation.

## Conclusions

The mortality due to COVID-19 is high among smokers than non-smokers with statistical significance. Thus, before admitting COVID-19 patients, to classify the patients as mild, moderate, and severe, the risk factor of the habit of smoking can be added. Even after admission, those patients with a history of smoking should be on continuous monitoring to minimize the adverse outcome. The majority of the patients who need hospital care were unvaccinated. Cigarette smoke is harmful to the lungs in a variety of ways, and further research is needed to understand why there is such a low proportion of current smokers among COVID-19 patients in hospitals. The impact of current smoking on SARS-CoV-2 infection is a delicate and complex topic that should be thoroughly investigated before sending out potentially misunderstood signals, and this study promotes further research.
